# Evaluating the Impact of Elective Nodal Irradiation for Dogs With Oral Malignant Melanoma Undergoing Hypofractionated Radiotherapy

**DOI:** 10.1111/vco.70005

**Published:** 2025-07-17

**Authors:** Patricia Gualtieri, Lisa Group, David M. Ruslander, Michael W. Nolan, Mary‐Keara Boss

**Affiliations:** ^1^ Department of Clinical Sciences, College of Veterinary Medicine and Biomedical Sciences Colorado State University Fort Collins Colorado USA; ^2^ Department of Environmental and Radiological Health Sciences, College of Veterinary Medicine and Biomedical Sciences Colorado State University Fort Collins Colorado USA; ^3^ BluePearl Pet Hospital Cary North Carolina USA; ^4^ Department of Clinical Sciences, College of Veterinary Medicine North Carolina State University Raleigh North Carolina USA

**Keywords:** dog, lymph node, melanoma, radiotherapy

## Abstract

Hypofractionated radiotherapy (hRT) is often used to treat dogs with oral malignant melanoma (OMM); however, there is no consensus as to whether clinically uninvolved regional lymph nodes should be prophylactically irradiated. The objective of this retrospective study is to compare outcomes for dogs with OMM treated with hRT+/– elective nodal irradiation (ENI). Dogs with nonmetastatic OMM undergoing hRT+/– ENI with a prescription of ≥ 30 Gy were included. Survival statistics were evaluated with Kaplan–Meier curves and log‐rank testing. Univariable and multivariable Cox proportional hazard models were used to assess how survival was impacted by the use of ENI, WHO T‐stage, mitotic count, RT technique, and use of Oncept melanoma vaccine. Data from four institutions and 100 dogs (80 with ENI and 20 without) were included. In the ENI group, nodal and distant metastases were documented in 4 and 30 dogs, respectively. In the non‐ENI group, nodal and distant metastases were documented in 6 and 4 dogs, respectively. There was no significant difference in the 1‐year nodal or distant progression‐free intervals (*p* = 0.174, and 0.563, respectively). The only variable maintaining significance on multivariable analysis was T‐stage (overall progression‐free survival, HR 1.393, *p* = 0.006; overall survival time, HR 1.426, *p* = 0.005; distant progression‐free interval, HR 1.521, *p* = 0.033). ENI did not measurably alter the oncologic outcomes in this study population. Results should be interpreted cautiously given the lack of standardised staging/restaging and the heterogenous nature of this clinical population. Future investigations are needed to clarify the role of ENI in the treatment of canine OMM.

## 1 Introduction

Elective nodal irradiation (ENI, also known as prophylactic nodal irradiation) is a technique that targets clinically uninvolved lymph nodes considered at risk of harbouring microscopic disease. The aim of ENI is to reduce the chance of locoregional failure and potentially increase overall survival time by reducing the likelihood of distant metastasis. While ENI may be logical in the setting of diseases with a high rate of occult micrometastatic nodal disease, there are also risks. Extending an RT field to cover regional lymph nodes increases the risk of morbidity. More concerning are emerging data from preclinical rodent tumour models suggesting that in certain disease settings (e.g., head and neck cancer), ENI may actually hasten metastatic tumour growth and decrease immune responses systemically [1, 2, 3].

In human cancer patients, the benefits of ENI may vary based on tumour location and patient characteristics. Phase 3 clinical trials have investigated the impact of ENI on distant metastasis and overall survival in patients with breast cancer. For example, the EORTC 22922/10925 trial identified a significant reduction in breast cancer mortality and recurrence with ENI of internal mammary and medial supraclavicular lymph nodes. However, this did not translate into improved overall survival [4]. Similarly, the KROG 08‐06 trial evaluated the addition of internal mammary node irradiation in node‐positive breast cancer patients. The results indicated that including internal mammary node irradiation did not significantly improve disease‐free survival, with the exception of a subgroup with medially or centrally located tumours [5]. These studies suggest that while ENI can reduce recurrence and breast cancer‐specific mortality, its effect on overall survival remains uncertain. In human head and neck carcinomas, regional metastasis represents a major prognostic factor [6]. This has prompted oncologists to electively irradiate lymph nodes on both sides of the neck as a longstanding practice [7, 8].

**FIGURE 1:**
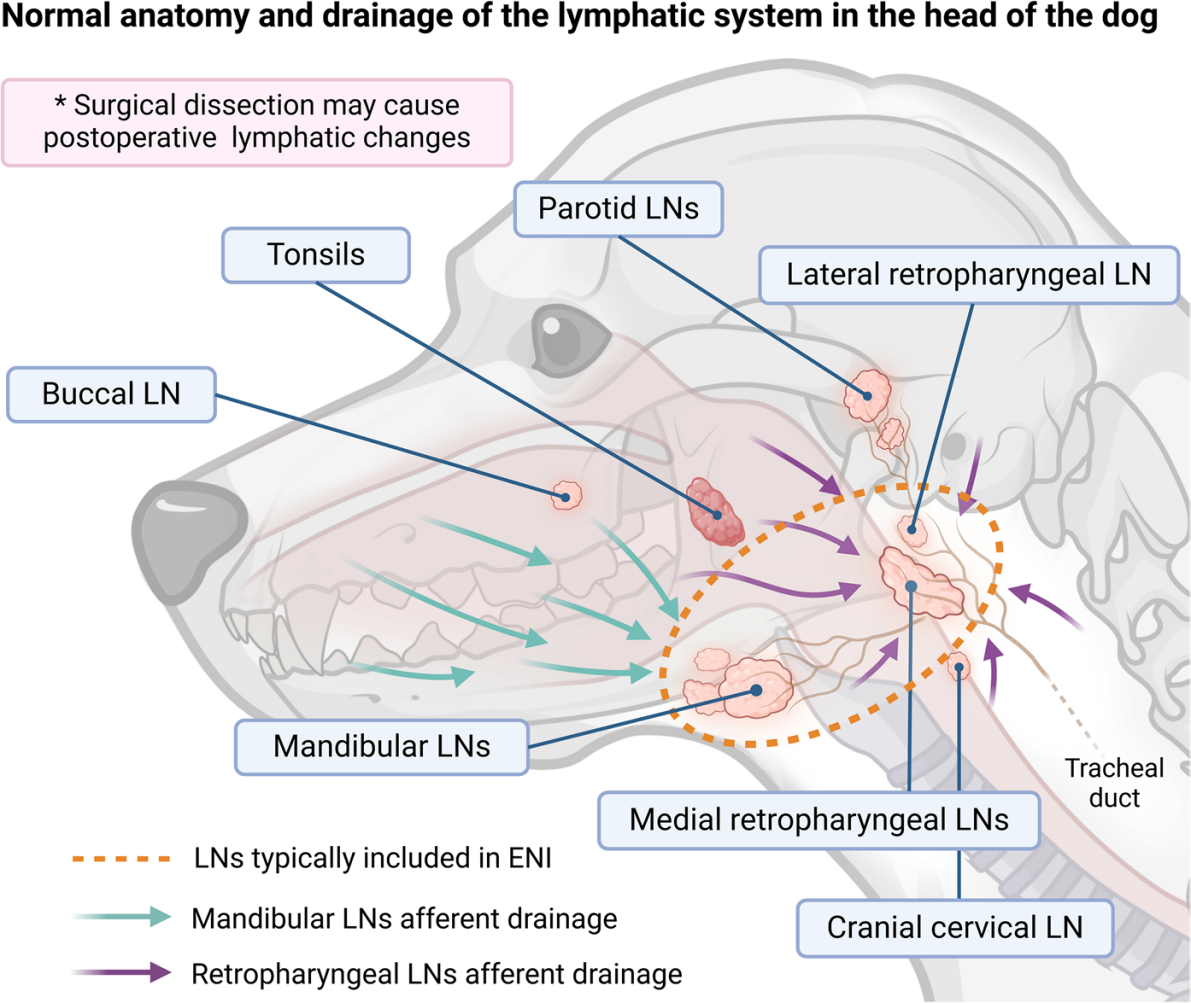
Lymphatic anatomy in the head of the dog and lymph node inclusion in elective nodal irradiation for canine oral tumours. Normal anatomic drainage is complex and may vary amongst patients. The afferent drainage to the mandibular lymph nodes typically includes the upper and lower lip, tip of the tongue, gums, cheek, hard and soft palate, nose, as well as various skull bones. The efferent vessels of the mandibular lymph nodes open into the ipsilateral or contralateral medial retropharyngeal lymph node and the lateral retropharyngeal lymph node, if present. The afferent drainage to the medial retropharyngeal lymph nodes includes the mid to caudal tongue, gingiva, hard and soft palate, tonsil, parotid, mandibular and sublingual salivary glands, pharynx and oesophagus, nasal cavity, larynx, thyroid, trachea (initial part) and ear, various skull bones as well as efferent vessels from the mandibular, buccal, parotid and lateral retropharyngeal and cranial cervical lymph nodes. The efferent vessels combine to form the tracheal duct [21, 22, 23]. Elective nodal irradiation for oral tumours in dogs typically aims to include unilateral [24] or bilateral mandibular and/or retropharyngeal lymph nodes [12, 13, 25]. Other lymph nodes in the vicinity, such as the cranial cervical lymph node, may be included in the RT field. Abbreviations: LN, lymph node; ENI, elective nodal irradiation.

The value of ENI has not been formally studied in veterinary oncology. In dogs with high‐grade mast cell tumours, elective nodal treatment via surgery or RT has been associated with prolonged progression‐free survival and overall survival [9, 10]. Likewise, ENI was reported to benefit cats with localised nasal lymphomas [11]. In dogs, ENI is often considered a component of care for canine oral malignant melanoma (cOMM) [11, 12, 13]. The rationale for ENI in the treatment of cOMM is that nodal metastasis is relatively frequent [14] and minimally invasive staging tests such as palpation [15, 16], imaging [17], and cytology [18, 19, 20] have variable accuracy for detecting occult disease. The normal anatomy of some dogs includes crossover of lymphatic drainage in the head and neck from one side to the other [21, 22, 23] and it is therefore unsurprising that dogs with OMM can experience contralateral and bilateral nodal metastases [14]; indeed this influences typical radiotherapy field designs as illustrated in Figure 1 [12, 13, 25]. Despite the frequency with which ENI is performed for canine OMM, its impact on clinical outcome remains uncharacterised. In a previous retrospective study of dogs with various oral tumours that had been treated with SBRT, ENI did not measurably alter the risk of developing regional lymph node metastasis [13]. This reference provided preliminary information in a smaller cohort of patients with mixed‐histotype oral tumours and variable nodal status, but a specific investigation into canine OMM was not performed. Therefore, in the current study, we build upon these preliminary findings by applying refined inclusion in an expanded cohort of canine OMM patients, including those with macroscopic or microscopic disease, treated with routinely used, veterinary hypofractionated, radiotherapy protocols.

## 2 Materials and Methods

### 2.1 Case Selection, Medical Record Data Acquisition and Treatment Planning Review

A multi‐institutional retrospective study was performed amongst four specialty veterinary centres in the United States. The search included client‐owned dogs histologically or cytologically diagnosed with OMM treated with hypofractionated radiotherapy (hRT), between December 2008 and April 2024. Inclusion criteria were a radiation prescription of ≥ 30 Gy total delivered in once or twice weekly fractions, with or without elective nodal irradiation of the bilateral mandibular and medial retropharyngeal lymph nodes. Dogs with microscopic and macroscopic primary disease burden were included. Complete staging, including lymph node mapping and sampling, was not required for inclusion in the study. Exclusion criteria were evidence of any metastasis at diagnosis, regional lymph node extirpation before RT, any prior RT for the OMM lesion, use of concurrent immunotherapy other than xenogeneic human tyrosinase (Oncept) and a follow‐up time of less than 6 months from the start of hRT.

Data collected included: patient demographics, date of diagnosis, medical and surgical management for the OMM prior to presentation for RT, staging modality, and results. The stage of the OMM was assigned according to the World Health Organization (WHO) TNM staging system [26]. Tumour characteristics recorded included specific tumour location (maxilla, mandible, lip/buccal, soft palate, tongue, other), tumour size obtained via calliper measurement of the longest tumour diameter on physical examination and/or electronic measurement of cross‐sectional imaging, disease burden at the time of presentation for RT, and pathology characteristics identified on cytology and/or histopathology reports. Information collected in the follow‐up period included improvement of clinical signs, development of adverse events, acute and late tissue toxicities, use of adjunctive systemic treatment such as chemotherapy or immunotherapy, dates and results of follow‐up staging via imaging, development of local, nodal or distant progressive disease based on medical record and communication log review, and date and cause of death. Acute effects were defined as toxicity signs observed within 90 days after treatment, and late effects were defined as toxicity signs noted > 90 days after treatment. Because radiation side effects had already been graded using the Veterinary Radiation Therapy Oncology Group (VRTOG v1.0) morbidity scoring scheme in a majority of cases, and full medical records were not available for review for these patients, toxicity was retrospectively graded using VRTOG v1.0, when not already documented, to maintain consistency [27].

Radiation treatment plans were assessed to obtain the following data: protocol, start and end dates of RT, completion of protocol (yes/no), total radiation dose, dose per fraction, RT technique categorised as computer‐based plan [intensity modulated RT (IMRT) or three‐dimensional conformal RT (3D‐CRT)] or manually calculated plan, radiation type (photons or electrons) and energy, use of bolus, wedges or field shaping devices, when recorded.

### 2.2 Statistical Analysis

Shapiro–Wilk test was used to test for normality. Fisher's exact test for categorical data, unpaired t‐test, and Mann–Whitney test for quantitative data were used to compare patient characteristics between groups. Outcomes were reported as overall progression‐free survival (OPFS), overall median survival time (OST), local progression‐free interval (L‐PFI), nodal progression‐free interval (LN‐PFI) and distant progression‐free interval (D‐PFI), and assessed using Kaplan–Meier curves and compared via log‐rank test. OPFS was calculated from the start of irradiation to the time of progression or death (censor = alive without progression or lost to follow‐up). OST was calculated from the start of irradiation to the time of death from any cause (censor = alive or lost to follow‐up). L‐PFI was calculated from the start of irradiation to the time of local progressive disease (PD) (censor = death with no local PD, alive or lost to follow‐up with no progression). LN‐PFI and D‐PFI were calculated from the start of irradiation to the time of development of nodal and distant metastasis, respectively (censor = death with no LN‐PD or D‐PD, alive or lost to follow‐up with no progression). The Kaplan–Meier method was used to estimate the 1‐year nodal and distant progression‐free interval proportions.

Univariable Cox proportional hazard models were used to estimate the hazard ratios of risk factors for OPFS, OST, LN‐PFI, and D‐PFI. Variables used in the analysis included use of ENI (yes/no), WHO T stage (tumour burden and size, T0‐T3), mitotic count (< 4 per 10 high‐powered field vs. ≥ 4 per 10 high‐powered field), use of Oncept vaccine (yes/no) and RT technique (manual/computerised). Multivariable Cox proportional hazard analysis was then performed and included any variable with: *p* < 0.2 on univariable analysis, direct relation to our primary study aim, or a known influence on survival as documented in prior peer‐reviewed literature. Statistical significance was set a priori at *p* < 0.05. All statistical analyses were performed using commercial software Prism v10.1.2 (GraphPad Software, San Diego, CA) and JMP Pro v18 (SAS Institute Inc., Cary, NC).

## 3 Results

### 3.1 Patient Demographics and Tumour Characteristics

A total of 100 dogs met the inclusion criteria. Treatment centres included Institution 1 (Varian Novalis TX; Varian Medical Systems Inc., Palo Alto, CA; *n* = 38 dogs), Institution 2 (Varian 2100C; *n* = 20 dogs), Institution 3 (prior to February 2021, a Varian 2100C/D was used, and a Varian Halcyon was used thereafter; *n* = 18 dogs) and Institution 4 (Varian Trilogy; Varian Medical Systems Inc., Palo Alto, CA; *n* = 24). Of these, 55% were cases that had been included in a previously published study [12]. Mixed breed dogs represented 21% of the study population; Labrador Retrievers (13%), Yorkshire Terriers (7%), Golden Retrievers (6%) and Pugs (5%) were the most common breeds in the study population. A total of 54% of dogs were male (intact *n* = 8, castrated *n* = 46) and 46% were female (all spayed).

The diagnosis of OMM was achieved via histopathology in 90% of cases, via cytology in 6%, and was not specified in 4% of the records. The tumour was incidentally identified on a routine annual exam or dental prophylaxis procedure in 31% of dogs. Specific tumour locations included maxilla (43%), mandible (34%), lip/buccal (8%), tongue (7%), soft palate (6%), tonsil (1%) and larynx (1%). Thoracic imaging was performed for all animals to rule out pulmonary metastasis, either via computed tomography (CT) (31%) or thoracic radiographs (44%), and modality was not specified in the remainder of cases (25%). Fine needle aspiration of at least one regional lymph node was performed in 72% of cases; the other 28% of cases lacked cytologic evaluation and were included on the basis of lymph nodes that appeared clinically normal. Abdominal staging with ultrasound or CT was conducted in 40% of dogs. None of the patients had evidence of nodal or distant metastatic disease at the time of diagnosis.

A total of 67% of dogs had at least one surgical procedure prior to RT. The intent was noted to be ‘curative’ (*n* = 26), ‘marginal’ (*n* = 15) or ‘unknown’ (*n* = 26). Margin status was available in 55/67 (82%) of the histopathology reports; nine cases were described as having ‘narrow’ or ‘complete’ margins, while the remainder were ‘incomplete’. Non‐steroidal anti‐inflammatories, including carprofen, piroxicam, meloxicam or grapriprant, were prescribed to 33% of animals before RT.

At the time of presentation for radiation therapy, 38% of patients had microscopic disease (T0), 5% were classified as T1, 27% as T2, and 15% as T3. Tumour stage was unknown in 15% of cases due to incomplete medical records. Demographics and tumour characteristics subdivided by treatment groups are summarised in Table 1. At the time of RT, there was no difference between the two study groups in terms of age (unpaired t‐test, *p* = 0.949), body weight (Mann–Whitney test, *p* = 0.789), sex (Fisher's exact test, *p* = 0.454), mitotic count (*p* = 0.403), stage at diagnosis (*p* = 0.540), T stage (*p* = 0.322) or tumour burden (*p* = 0.208). A significantly higher proportion of dogs received treatment with a computerised plan in the ENI group (Fisher's exact test, *p* = 0.035).

**TABLE 1:** Summary of patient demographics and tumour characteristics amongst the two groups in the study population.

	No LN RT (*n* = 20)	ENI (*n* = 80)	*p*
Age (years)	11.3 ± 2.7	11.3 ± 2.3	0.949
Weight (kg)	24.5 (1.7–44)	23.1 (2.2–55.2)	0.789
Sex			
Male	9 (45%)	45 (56.25%)	0.454
Female	11 (55%)	35 (43.75%)	
Mitotic count			
≥ 4/10 hpf	15 (75%)	58 (72.5%)	0.403
< 4/10 hpf	3 (15%)	6 (7.5%)	
Unknown	2 (10%)	16 (20%)	
WHO stage at diagnosis			
Stage I	3 (15%)	18 (22.5%)	0.540
Stage II	8 (40%)	30 (37.5%)	
Stage III	5 (25%)	12 (15%)	
Unknown	4 (20%)	20 (25%)	
T stage at time of RT			
T0	5 (25%)	33 (41.25%)	0.322
T1	1 (5%)	4 (5%)	
T2	7 (35%)	20 (25%)	
T3	5 (25%)	10 (12.5%)	
Unknown	2 (10%)	13 (16.25%)	
Tumour burden			
Macroscopic	15 (75%)	47 (58.75%)	0.208
Subclinical	5 (25%)	33 (41.25%)	
RT technique			
Manual	11 (55%)	23 (28.75%)	**0.035**
Computerised	9 (45%)	57 (71.25%)	

*Note:* Highlighted in bold significance for *p* < 0.05.

Abbreviations: ENI, elective nodal irradiation; No LN RT, no lymph node irradiation; RT, radiation therapy.

### 3.2 Radiotherapy Planning and Treatment

For each individual dog, the design of the irradiation protocol (e.g., radiation dose, fractionation, whether or not to include lymph nodes) was at the discretion of the attending radiation oncologist. Radiation dose varied from 5 to 9 Gy per fraction, with either 4 once‐weekly fractions, or 6 delivered once or twice per week. Protocols included 6 Gy × 6 fractions (*n* = 80 dogs), 9 Gy × 4 (9 dogs), 8 Gy × 4 (8 dogs), 6 Gy × 5 (1 dogs), 5 Gy × 6 (1 dogs), and one dog received an initial dose of 6 Gy followed by three fractions of 8 Gy. A total of 80 dogs underwent ENI and 20 did not receive any lymph node irradiation (‘no LN RT’). Treatment plans were manually calculated for 34 dogs, utilising electrons in two cases and 6 MV photons in the remainder. Computerised plans were created via 3D‐CRT in 44 dogs, using 6 MV photons (*n* = 41), 10 MV photons (*n* = 1), or a combination of both energies (*n* = 2). 6 MV photons were used for IMRT (*n* = 22). Tissue‐equivalent bolus material was used either on the skin or in the oral cavity for dose buildup in 71 cases. Wedges were used in 12 plans. Field shaping devices included static multi‐leaf collimators (MLC) (*n* = 35), dynamic MLC/IMRT (*n* = 22) and blocks (*n* = 1). Nodal treatment planning information was available for 59 of the 80 cases treated with ENI. A separate RT plan or treatment isocenter was used for lymph node irradiation in 4/59 (6.77%) of cases. In all 59 plans, the RT prescription for the ENI was the same as for the primary tumour site. A total of 10 dogs in the study failed to complete the prescribed treatment protocol but were included in this intent‐to‐treat analysis.

### 3.3 Patient Follow‐Up, Radiation‐Associated Toxicity and Oncologic Outcomes

At the time of data collection, 76 of the 100 included dogs were deceased, 4 were alive, and 20 had been lost to follow‐up. The median follow‐up time for the patients censored from the survival analysis was 364 days (range: 181–1041). When evaluating the cause of death, a total of 42/76 (55.26%) dogs died or were euthanised due to their OMM, 19/76 (25%) were euthanised due to unrelated causes, and the reason was unknown in the remainder of the cases. No dogs were reported to die or be euthanised due to radiation toxicity. Ten dogs were euthanised within 3 months of starting RT.

Adjunctive systemic therapy (chemotherapy or immunotherapy) was recorded in 65% of animals. A total of 58 dogs received Oncept; of these, 48 received ENI (i.e., 60% of all ENI group) and 10 did not (i.e., 50% of the No LN RT group). A total of 22 dogs received at least one dose of chemotherapy (carboplatin *n* = 19, toceranib *n* = 6, temozolomide *n* = 2, dacarbazine *n* = 1, cyclophosphamide *n* = 1, chlorambucil *n* = 1, bleomycin electrochemotherapy *n* = 1, and one dog received multidrug protocol for development of epitheliotropic lymphoma).

Acute radiation toxicities were recorded in 56/83 (67.46%) of dogs that had been assessed within 3 months after RT. Of these recorded acute toxicities, 51 dogs had been treated with ENI and five dogs had no LN RT. A total of 52 dogs (62.65%) developed grade 1–2 acute skin and/or mucosal toxicity; one dog (1.2%) developed grade 2 eye toxicity (bilateral KCS); three dogs (3.61%) developed grade 3 skin and/or mucosal toxicity, and of these, one dog experienced mucositis, edema of muzzle and lips, as well as severe pain requiring G‐tube placement and hospitalisation. Late adverse events were recorded in 17/76 (21%) dogs assessed 3 months or more after RT. Of these, 15 were in the ENI group and 2 in the no LN RT group. These included five cases (6.57%) of grade 3 bone toxicity (osteoradionecrosis and fistula formation), four dogs (5.26%) were recorded to develop grade 1 skin toxicity (alopecia and hyperpigmentation), two dogs (2.63%) developed grade 1–2 eye toxicity (keratoconjunctivitis sicca) and one case (1.31%) was reported to have a grade 1 CNS toxicity (head tilt) possibly related to radiation therapy; other adverse events not fitting VRTOG v1.0 criteria included chronic mucosal ulceration (*n* = 2, 2.63%), xerostomia (*n* = 2, 2.63%), devitalized tooth (*n* = 1, 1.31%). Both cases of xerostomia occurred in dogs that received ENI; one had a manually calculated plan and one had a 3D‐CRT plan. The full study animal population demographics, RT plans, and dosimetry, as well as follow‐up data are available in Appendix S1.

Nodal metastasis was documented after RT in 10 dogs, including 6 from the ENI group and 4 from the no LN RT group (i.e., 7.5% and 20% of those groups, respectively). The 1‐year lymph node progression‐free survival rate was 90.15% in the ENI group (*p* = 0.174, 95% CI: 0.4403 to 15.33) and 81.41% in the No LN RT group. Distant progressive disease (D‐PD) was documented on thoracic imaging, when performed, after hRT in 34 dogs, and of these, 30 were in the ENI group and 4 in the No LN RT group. The 1‐year distant progression‐free survival rate was 63.23% in the ENI group (*p* = 0.563, 95% CI: 0.2810–1.908) and 72.39% in the No LN RT group.

**FIGURE 2:**
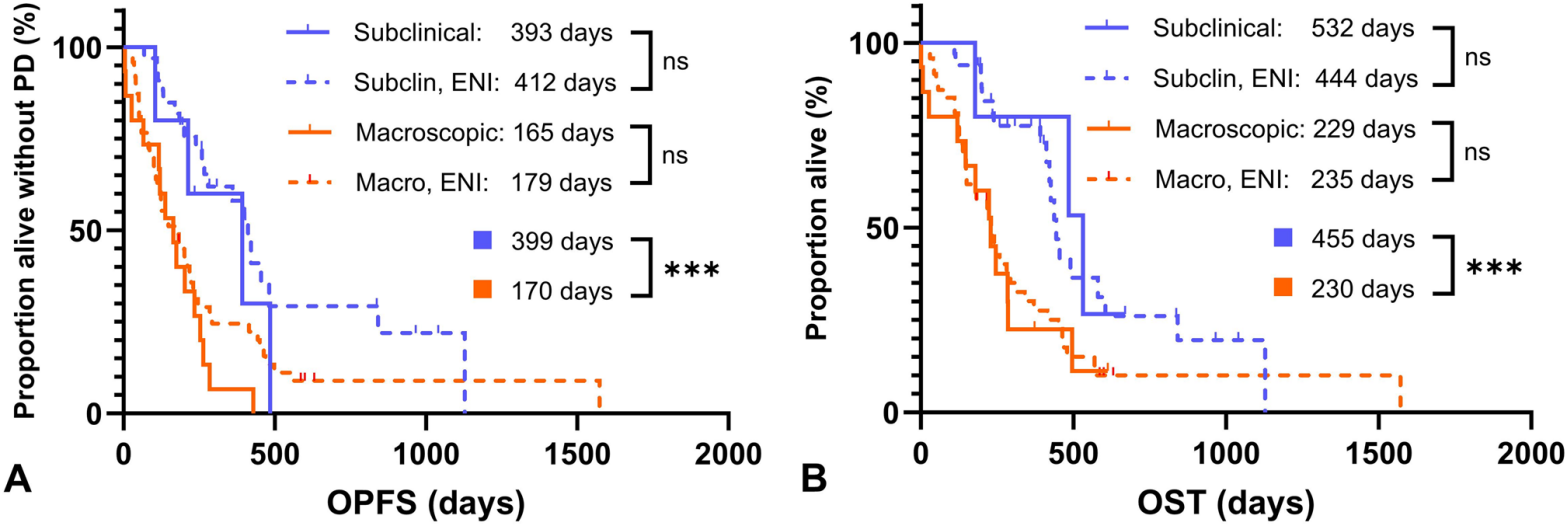
Outcomes of 100 dogs with oral malignant melanoma treated with hypofractionated RT with or without elective nodal irradiation, based on disease burden. (A) There was no difference in OPFS with the use of ENI when comparing dogs in the subclinical (log‐rank test, *p* = 0.36, 95% CI: 0.3260–2.791) and macroscopic (*p* = 0.20, 95% CI: 0.5121–1.659) disease settings. There was a significant difference between disease burden groups (*p* = 0.0004). (B) There was no difference in OST with the use of ENI when comparing dogs in the subclinical (log‐rank test, *p* = 0.82, 95% CI: 0.3546–4.049) and macroscopic (*p* = 0.83, 95% CI: 0.5551–2.068) disease settings. There was a significant difference between disease burden groups (*p* = 0.0006). Tick marks on the Kaplan–Meier curves represent censored patients. PD, progressive disease.

The median OPFS time was 224 days (95% CI: 179–267 days), and the median OST was 294 days (95% CI: 236–426 days). Univariable and multivariable Cox proportional hazard analysis results are summarised in Table 2. Although the use of ENI and Oncept were significantly associated with improved outcome on univariable analysis, on multivariable analysis, T‐stage was the only risk factor maintaining statistical significance for OPFS time (HR: 1.393, 95% CI: 1.197–1.769, *p* = 0.006), OST (HR: 1.42, 95% CI: 1.110–1.832, *p* = 0.005), and D‐PFI (HR: 1.521, 95% CI: 1.036–2.266, *p* = 0.033) (Table 3). Overall median L‐PFI was 444 days (95% CI: from 264 to not reached) for all dogs. When evaluating disease burden, dogs with macroscopic tumours had a median L‐PFI of 429 days (95% CI: 230–469 days), while for dogs with subclinical disease the median L‐PFI was not reached (log‐rank, *p* = 0.07). Outcomes for the total study population based on disease burden and nodal irradiation approach are summarised in Figure 2A,B.

**TABLE 2:** Univariable and multivariable Cox proportional hazard ratio analysis for outcome data of dogs with oral malignant melanoma treated with hypofractionated radiotherapy.

(a) Univariable Cox proportional HR analysis												
	OPFS			OST			LN‐PFI			D‐PFI		
	HR	95% CI	*p*	HR	95% CI	*p*	HR	95% CI	*p*	HR	95% CI	*p*
T stage	1.517	1.237–1.864	**< 0.0001**	1.529	1.229–1.907	**0.0001**	1.163	0.6342–2.051	0.6034	1.674	1.196–2.375	**0.0030**
MC	1.461	0.3646–7.229	0.6057	0.4858	0.1849–1.059	0.0989	0.5575	0.02963–3.163	0.5867	0.1736	0.009–0.8340	0.0879
ENI	0.5710	0.344–0.9886	**0.0360**	0.8580	0.4981–1.574	0.5991	0.3478	0.09929–1.361	0.1020	1.734	0.6816–5.850	0.3026
XRT Tx	0.9476	0.6005–1.531	0.8206	0.8809	0.5462–1.455	0.6102	0.2949	0.07535–1.033	0.0587	1.126	0.5496–2.480	0.7554
Oncept	0.7093	0.4537–1.113	0.1320	0.6153	0.384–0.9886	**0.0432**	0.3916	0.09916–1.385	0.1498	0.7266	0.3641–1.470	0.3649

(b) Multivariable Cox proportional hazard ratio analysis												
	OPFS			OST			LN‐PFI			D‐PFI		
	HR	95% CI	*p*	HR	95% CI	*p*	HR	95% CI	*p*	HR	95% CI	*p*
T stage	1.393	1.097–1.768	**0.0063**	1.426	1.110–1.832	**0.0053**	0.9613	0.4659–1.840	0.9077	1.521	1.036–2.266	**0.0336**
MC	0.5274	0.1785–1.252	0.1887	0.6030	0.2012–1.464	0.3078	0.6679	0.03437–4.144	0.7150	—	—	> 0.9999
ENI	1.543	0.7952–2.839	0.1785	0.8364	0.3925–1.636	0.6205	2.000	0.3802–8.799	0.3685	0.4830	0.1117–1.456	0.2499
XRT Tx	1.283	0.7230–2.353	0.4058	1.068	0.5891–2.012	0.8314	0.4744	0.1072–2.099	0.3074	1.360	0.5380–3.884	0.5340
Oncept	0.7298	0.4129–1.290	0.2761	0.6188	0.3417–1.118	0.1101	0.7538	0.1708–3.379	0.7003	0.4128	0.1606–1.000	0.0542

*Note:* (a) In the univariable analysis, information was available for all cases regarding the use of elective nodal irradiation, radiation treatment technique and Oncept, while observations were limited to *n* = 85 dogs for T stage and *n* = 82 dogs for mitotic count. (b) The number of observations included in the outcome analysis was limited to *n* = 72 dogs, due to missing data. Tumour T stage (WHO T0–T3) represents primary tumour disease burden and size at the time of RT presentation, not at the time of diagnosis. Highlighted with bold significance for *p* < 0.05.

Abbreviations: D‐PFI, distant progression free interval; ENI, elective nodal irradiation; LN‐PFI, nodal progression free interval; MC, mitotic count; OPFS, overall progression free survival; OST, overall median survival time; XRT Tx, radiotherapy technique (computer‐based vs. manual calculation).

**TABLE 3:** Summary of patient outcome based on WHO T stage at the time of radiation treatment.

Days	T0 (*n* = 38)		T1 (*n* = 5)		T2 (*n* = 27)		T3 (*n* = 15)	
	ENI (*n* = 33)	No LN RT (*n* = 5)	ENI (*n* = 4)	No LN RT (*n* = 1)	ENI (*n* = 20)	No LN RT (*n* = 7)	ENI (*n* = 10)	No LN RT (*n* = 5)
OPFS	412	393	243	235	137	203	151	120
	(259–482)*	(105–485)	(77–613)		(83–218)*	(117–264)	(41–246)*	(2–264)
OST	444	532	288	246	218	285	207	120
	(412–605)*	(168–669)	(142–631)		(126–464)*	(179–613)	(41–246)*	(2–496)

*Note:* This information was calculated via Kaplan–Meier curve for 85 dogs with available information. Days reported in median and (95% CI or range; * indicates CI).

A separate statistical analysis was conducted to investigate the effects of ENI on a subgroup of study subjects with microscopic and/or low‐stage (I/II) disease, where the reported median OSTs are > 14 months and prognosis is often driven by distant metastasis [12, 28, 29]. When dogs with macroscopic tumours of unknown size (*n* = 15) or stage III disease (*n* = 15) were excluded, there was no detectable difference in either OPFS time (*p* = 0.16, 95% CI: 0.3045 to 1.336) or OST (*p* = 0.97, 95% CI: 0.7135–3.051) between groups (ENI vs. no), and no risk factors for early oncologic failure were identified on Cox proportional hazard analysis. Results were similar when an additional 17 dogs with lack of regional lymph node sampling were excluded. This information is summarised in Appendix S2, Subgroup Analysis.

## 4 Discussion

This study was performed to investigate the potential impacts of ENI on oncologic outcomes of dogs with OMM treated with routinely used hypofractionated radiotherapy protocols. The OPFS and OST were comparable to those previously reported for dogs with OMM treated with RT [12, 25, 28, 29, 30, 31]. The results of the study confirm the widely accepted knowledge that primary tumour size and disease burden at the time of RT are the most important prognostic factors for dogs with OMM when there is no evidence of metastasis [12, 24, 28, 29, 31]. In this study, the use of the Oncept vaccine was not a significant predictor of outcome; however, it did approach significance with a protective effect for D‐PFI (HR: 0.412, 95% CI: 0.1606–1.000, *p* = 0.054). Unfortunately, due to the retrospective nature of the study and lack of complete physical exam findings, radiographic studies and/or cross‐sectional imaging for all cases, additional factors that have shown to be prognostic, such as tumour location [12, 28], presence of bony lysis [12], as well as primary tumour response to treatment [24, 30], could not be investigated. The inconsistent use of systemic chemotherapy in the follow‐up period, often prescribed at the time of disease progression, affected the ability to conduct meaningful investigations of the role of chemotherapy within the current study population. With regard to our primary hypothesis, ENI was associated with improved OPFS on univariable analysis, but when accounting for other factors in the multivariable models and subgroup assessments, ENI was not significantly associated with measurable benefit or detriment. Interestingly, in treating human head and neck mucosal melanoma with elective nodal treatment in the form of either ENI or elective nodal dissection, there is no definitive evidence of clinical benefit or detriment [32, 33, 34, 35, 36, 37].

One limitation of this research is that nodal and distant metastases were relatively infrequently detected after RT. This could be due to (1) missing data because of the lack of standardised re‐staging, or (2) the ENI that was conducted in the majority of study animals effectively prevented distant and/or nodal/regional metastasis from occurring. Nonetheless, the proportion of dogs that were metastasis‐free at 1‐year post‐RT was not significantly different amongst study groups. While the T stage represented the only risk factor for distant disease progression, it was not associated with the development of nodal metastasis.

In this study, severe toxicities such as acute ulceration and necrosis of oral mucosa and skin, osteoradionecrosis and fistula formation were uncommon and not attributable to the use of ENI. Interestingly, xerostomia, which is rarely reported in veterinary medicine [38, 39] was seen in two dogs that received ENI in this study. It is likely that ENI of cervical lymph nodes could increase the risk for xerostomia, as the irradiated nodes are in close proximity to the major salivary glands; however, the use of highly conformal RT, such as IMRT, could reduce this risk in canine patients with oral tumours undergoing ENI [25]. Due to limitations of the retrospective evaluation of acute and late toxicities of the dogs in this study, we are unable to compare the toxicity profiles of dogs treated with ENI versus No LN RT. There was no notable difference in the acute and late toxicities between the two treatment groups. For this analysis, VRTOG v1.0 [27] was used for toxicity reporting due to the majority of animals being treated and toxicity recorded before the VRTOG v2.0 [40] was published. This may have underestimated the detection and reporting of radiation‐induced side effects, in addition to the limitations associated with the retrospective nature of the data.

While this work represents preliminary data that is intended to encourage and stimulate further investigation, it also raises important questions regarding the underlying reasons for the lack of clinical benefit or detriment. For dogs with locally advanced disease, this can be reasonably explained by the fact that local progressive disease represents the main cause for early progression and death. However, the matter remains open for the dogs with subclinical melanoma that underwent treatment, as their prognosis is better, with OST greater than 14 months [12, 29]. Putting aside the concern that failure to measure an impact (positive or negative) from ENI may reflect low statistical power, there is also a fundamental question that could be asked about the biological role of lymph nodes and lymphatics in the process of cancer metastasis. Draining lymph nodes are undoubtedly a sentinel that carries important prognostic information in cancer staging: in other words, when LNs are identified as cancer‐cell positive, they represent a red flag for aggressive tumour behaviour. However, it is unclear whether lymph nodes harbouring disease, either microscopic or overtly infiltrated, play an active or passive role in cancer dissemination [41]. Do they represent an “immune station” that can filter metastasising cells and function as an active reservoir of tumour cells that are ready to take the next step and disseminate systemically through lymphatics? Do they provide a highway that can be taken as a parallel path to hematogenous dissemination? Preclinical mouse models have shown that lymph node metastases can be a source of cancer cells for distant metastases and lymph node blood vessels can serve as an exit route for systemic dissemination [42, 43]. Or are they a passive strainer that can capture subcellular debris and cells shed by normal tissues as well as cancer cells that venture no further? The lack of overt clinical benefit in a number of studies for human and canine elective nodal treatment for different tumour types [41], including melanoma in humans [33, 34] and dogs [18], would support the latter idea. Perhaps all these processes could occur during cancer dissemination, depending on the degree of nodal infiltration, as observed with the histological node classification in mast cell tumour disease in dogs [44], or may occur differently when considering different tumour types and species.

There is growing evidence for the role of RT to modulate the tumour microenvironment, increase local and systemic anti‐tumour immunity, and, RT in combination with immunotherapy, particularly immune checkpoint inhibitors, may have the potential to improve therapeutic outcomes for cancer patients [1, 45, 46]. There is increasing clinical interest in investigating radio‐immunotherapy combinations in both canine [28, 46, 47] and human patients [37, 48]. However, there is also emerging preclinical data highlighting the concern that concurrent ENI with tumour irradiation mitigates these systemic and local immune responses, specifically due to effects associated with effector CD4 and CD8 T cells [1, 49]. While the preclinical data suggest that concurrent ENI disrupts the positive results associated with radiation and immunotherapy [1, 3], delayed (adjuvant), but not neoadjuvant, draining lymph node irradiation overcomes these detrimental effects, allowing the induction of long‐lasting tumour‐specific immunity while achieving nodal disease control [1, 2]. If similar ENI effects were to be recapitulated in dogs with naturally occurring OMM, this would have important therapeutic implications and would highlight the need for careful considerations for optimisation of ENI with combinatorial therapy, particularly as immune checkpoint inhibitors specific for canine patients (e.g., gilvetmab) are becoming available worldwide for research and commercial use [46, 50]. Future efforts for the treatment of cOMM should focus on strategizing the timing of local, regional, and systemic therapy combinations to optimise tumour control, enhance anti‐tumour responses, and preserve normal tissue health. These preliminary data altogether did not show any obvious benefit or detriment with the use of ENI. Additional research is needed.

## 5 Limitations

Additional limitations of this study are mostly associated with the retrospective nature of the data, which has affected the ability to provide complete information regarding tumour characteristics (such as size, location, bony lysis, histopathology features, etc.) and patient staging at presentation, plan parameter characteristics and dosimetry, evaluation of tumour response and toxicity in the follow‐up time, as well as detection of loco‐regional and distant metastatic disease. Due to the retrospective and multi‐institutional case selection, there is heterogeneity of protocol prescription in terms of fraction size, total dose, time to complete delivery of protocol, lack of standardised re‐staging and post‐RT medical management, which may have affected outcome and/or development of side effects.

## 6 Conclusion

In this study population, ENI provided no measurable benefit or detriment in terms of toxicity or survival. Our failure to provide evidence for or against ENI likely reflects statistical power that was compromised by a highly heterogeneous study population. To better clarify the utility of ENI, further research is needed. This might ideally be in the form of prospective, randomised clinical trials with refined inclusion criteria. For example, focusing on dogs with low‐stage disease might be ideal since overall survival in those cases is often driven by distant metastasis versus dogs with large primary tumours, where local tumour control is frequently the determinant of survival. It would also be important to utilise more comprehensive lymph node staging, which could be achieved via sentinel lymph node mapping or routine pathologic evaluation of the bilateral mandibular and medial retropharyngeal lymph nodes.

## Ethics Statement

Ethical review and approval were not required due to the retrospective nature of the study involving client‐owned dogs that had previously consented to anaesthesia and radiotherapy. Written informed consent was not obtained for the study purposes, as existing clinical data were used retrospectively.

## Conflicts of Interest

The authors declare no conflicts of interest.

## Supporting information


**Appendix S1.** Supporting Information.

**Appendix S2.** Supporting Information.

## Data Availability

The data that supports the findings of this study are available in the Supporting Information of this article.
