# Supplementary material for: Evaluating the Impact of Elective Nodal Irradiation for Dogs With Oral Malignant Melanoma Undergoing Hypofractionated Radiotherapy
Source: Vet Comp Oncol. 2025 Jul 17;23(4):518–27. doi: 10.1111/vco.70005 (PMC12617689; doi:10.1111/vco.70005)
Supplement: Supplementary file 2 — Appendix S2. Supporting Information. [file VCO-23-518-s001.docx]

Table S1a. Univariable Cox proportional HR analysis

|  | **OPFS** (n = 70) | | | **OST** (n = 70) | | |
| --- | --- | --- | --- | --- | --- | --- |
|  | HR | 95% CI | P value | HR | 95% CI | P value |
| **T stage** | 1.552 | 1.163 to 2.071 | **0.0027** | 1.451 | 1.067 to 1.968 | **0.0164** |
| **MC** | 2.428 | 0.8746 to 10.08 | 0.1407 | 2.147 | 0.7675 to 8.948 | 0.2065 |
| **ENI** | 0.6265 | 0.3343 to 1.261 | 0.1635 | 0.9866 | 0.4950 to 2.188 | 0.9713 |
| **XRT Tx** | 1.014 | 0.5581 to 1.783 | 0.9626 | 1.212 | 0.6482 to 2.198 | 0.5347 |
| **Oncept^TM^** | 0.6846 | 0.3910 to 1.203 | 0.1836 | 0.5725 | 0.3139 to 1.044 | 0.0669 |

Table S1b. Multivariable Cox proportional HR analysis

|  | **OPFS** (n = 61) | | | **OST** (n = 61) | | |
| --- | --- | --- | --- | --- | --- | --- |
|  | HR | 95% CI | P value | HR | HR | 95% CI |
| **T stage** | 1.291 | 0.9254 to 1.801 | 0.1304 | 1.267 | 0.8936 to 1.794 | 0.1797 |
| **MC** | 2.079 | 0.7020 to 8.902 | 0.2420 | 1.763 | 0.5898 to 7.578 | 0.3670 |
| **ENI** | 0.6212 | 0.3173 to 1.293 | 0.1803 | 1.001 | 0.4858 to 2.268 | 0.9986 |
| **XRT Tx** | 0.7462 | 0.3807 to 1.406 | 0.3762 | 1.023 | 0.5082 to 1.980 | 0.9478 |
| **Oncept^TM^** | 0.6603 | 0.3527 to 1.233 | 0.1909 | 0.5699 | 0.2956 to 1.091 | 0.0888 |

**Table S1: Univariable and multivariable Cox Proportional Hazard Ratio analysis for outcome data of dogs with early stage-disease oral malignant melanoma treated with hypofractionated radiotherapy.** In this study subgroup, dogs with WHO TNM stage III disease or unknown macroscopic tumor size were excluded, leaving 70 dogs for the analysis. Table S1a: In the univariable analysis, information was available for all cases in terms of use of elective nodal irradiation, radiation treatment technique and Oncept^TM^, while observations were limited to n = 61 dogs for mitotic count. Table S1b: the number of observations included in the outcome analysis are in parenthesis. Tumor T stage (WHO T0 to T3) represents primary tumor disease burden and size at time of RT presentation, not at time of diagnosis. Highlighted significance for p < 0.05. *Abbreviations:* OPFS, overall progression free survival; OST, overall median survival time; LN-PFI, nodal progression free interval; D-PFI, distant progression free interval; MC, mitotic count; ENI, elective nodal irradiation; XRT Tx, radiotherapy technique (computer-based vs manual calculation).

Table S2a. Univariable Cox proportional HR analysis

|  | **OPFS** (n = 53) | | | **OST** (n = 53) | | |
| --- | --- | --- | --- | --- | --- | --- |
|  | HR | 95% CI | P value | HR | 95% CI | P value |
| **T stage** | 1.552 | 1.123 to 2.137 | **0.0070** | 1.451 | 1.067 to 1.968 | **0.0164** |
| **MC** | 3.303 | 0.9902 to 20.49 | 0.1033 | 2.615 | 0.7844 to 16.22 | 0.1897 |
| **ENI** | 0.6756 | 0.3237 to 1.587 | 0.3269 | 1.071 | 0.4768 to 2.860 | 0.8783 |
| **XRT Tx** | 1.175 | 0.5962 to 2.214 | 0.6272 | 1.417 | 0.7071 to 2.737 | 0.3086 |
| **Oncept^TM^** | 0.7132 | 0.3791 to 1.358 | 0.2955 | 0.6027 | 0.3107 to 1.178 | 0.1331 |

Table S2b. Multivariable Cox proportional HR analysis

|  | **OPFS** (n = 47) | | | **OST** (n = 47) | | |
| --- | --- | --- | --- | --- | --- | --- |
|  | HR | 95% CI | P value | HR | HR | 95% CI |
| **T stage** | 1.286 | 0.8904 to 1.839 | 0.1706 | 1.357 | 0.9292 to 1.964 | 0.1064 |
| **MC** | 2.870 | 0.7986 to 18.37 | 0.1651 | 2.343 | 0.6514 to 14.98 | 0.2619 |
| **ENI** | 0.6775 | 0.3112 to 1.636 | 0.3512 | 1.197 | 0.5127 to 3.271 | 0.6982 |
| **XRT Tx** | 1.001 | 0.4603 to 2.042 | 0.9975 | 1.475 | 0.6615 to 3.124 | 0.3210 |
| **Oncept^TM^** | 0.5965 | 0.2936 to 1.214 | 0.1499 | 0.5045 | 0.2366 to 1.067 | 0.0723 |

**Table S2: Univariable and multivariable Cox Proportional Hazard Ratio analysis for outcome data of dogs with early stage-disease staged with regional lymph node aspirate(s).** In this study subgroup, dogs with WHO TNM stage III disease, unknown macroscopic tumor size and/or lacked staging with at least one lymph node aspirate were excluded, leaving 53 dogs for the analysis. Table S1a: In the univariable analysis, information was available for all cases in terms of use of elective nodal irradiation, radiation treatment technique and Oncept^TM^, while observations were limited to n = 47 dogs for mitotic count. Table S1b: the number of observations included in the outcome analysis are in parenthesis. Tumor T stage (WHO T0 to T3) represents primary tumor disease burden and size at time of RT presentation, not at time of diagnosis. Highlighted significance for p < 0.05. *Abbreviations:* OPFS, overall progression free survival; OST, overall median survival time; LN-PFI, nodal progression free interval; D-PFI, distant progression free interval; MC, mitotic count; ENI, elective nodal irradiation; XRT Tx, radiotherapy technique (computer-based vs manual calculation).
